# A successful combined laparoscopic cholecystectomy and laparoscopic exploration of common bile duct for acute gangrenous cholecystitis and choledocholithiasis during pregnancy: A case report

**DOI:** 10.1016/j.ijscr.2019.03.062

**Published:** 2019-04-08

**Authors:** Si-Yuan Qiu, Kelvin K. Ng, Tan-To Cheung, Chun-Hong Liu, Hong-Tao Zhu, Bang-Ren Xu, Ren Ji, Chung-Mau Lo

**Affiliations:** a Department of Surgery, The University of Hong Kong - Shenzhen Hospital, Shenzhen, China; b Department of Surgery, University of Hong Kong, Hong Kong, China

**Keywords:** Pregnancy, Laparoscopic exploration of common bile duct, Choledocholithiasis, Case report

## Abstract

**Introduction:**

Choledocholithiasis during pregnancy is relatively uncommon but it can be life-threatening. Therapeutic endoscopic retrograde cholangiopancreatography (ERCP) poses the risk of ionizing radiation exposure to the developing fetus. Other strategies are thus needed to tackle this problem.

**Presentation of case:**

A 38-year-old 8 weeks’ pregnant lady presented with acute onset of right upper quadrant pain. Ultrasonography showed features of acute cholecystitis and choledocholithiasis, which was later confirmed by magnetic resonance cholangiogram. Emergency combined laparoscopic cholecystectomy (LC) and laparoscopic exploration of common bile duct (Lap ECBD) were performed. Lap ECBD through trans-cystic duct approach was adopted. The patient recovered uneventfully and was discharged on postoperative day 4. A healthy baby boy was delivered at 40 weeks’ gestation without developmental problem.

**Conclusion:**

Lap ECBD avoided ionizing radiation for choledocholithiasis during pregnancy, and can combined with LC as an effective minimally invasive procedure for complicated gallstone disease.

## Highlights

• Complicated gallstone disease during pregnancy can be successfully managed by combined laparoscopic cholecystectomy and exploration of common bile duct through trans-cystic duct approach.
• This approach is safe and can cure cholecystitis and choledocholithiasis in one goal.
• This approach avoids ionizing radiation to the developing fetus.

## 1 Introduction

The incidence of gallstone-related complications in pregnant woman is low (0.05 to 0.33%) [1,2]. Serious complications of gallstones, including acute cholecystitis, choledocholithiasis, and acute pancreatitis, account for less than 10% of all pregnant patients with symptomatic gallstone disease [[3], [4], [5]]. Indications for operation include repeated biliary colic or severe complications of gallstone disease. Management of choledocholithiasis during pregnancy requires special attention since the widely accepted therapeutic endoscopy (endoscopic retrograde cholangiopanceatography, ERCP) may pose ionized irradiation to the developing fetus. Alternative strategies are thus necessary to tackle this clinical situation. These include non-irradiation ERCP and laparoscopic exploration of common bile duct (lap CBD). Successful cases of lap ECBD are rarely reported in the literature. We herein reported a successful combined laparoscopic cholecystectomy (LC) and Lap ECBD for gangrenous cholecystitis and choledocholithiasis in a 8 weeks’ pregnant patient. This case has been made according to the SCARE criteria [6].

## 2 Case presentation

**Fig. 1:**
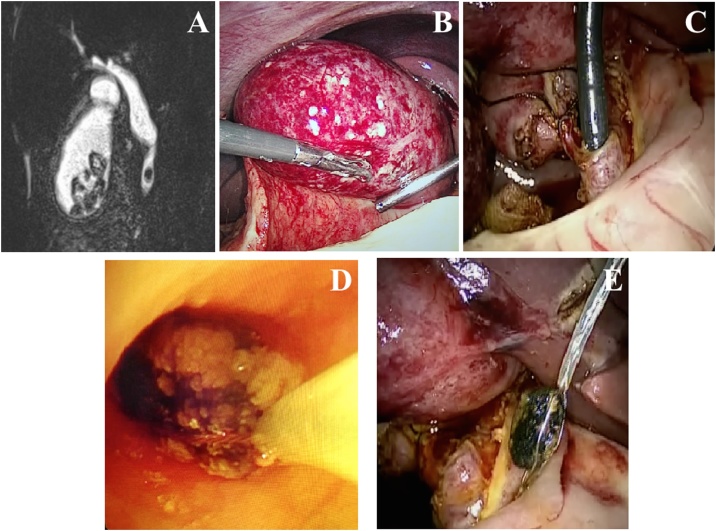
(A): MRCP showed a CBD stone in the lower CBD and multiple stones in gallbladder. (B): Fundus and body of gallbladder were gangrenous. (C): Laparoscopic transcystic duct choledochoscopy. (D): Removed the CBD stone by basket through choledochoscopy. (E): Transcystic duct CBD stone extraction by basket through choledochoscopy.

A 38-year-old pregnant woman (8 weeks’ gestation) presented with acute onset right upper quadrant pain. On abdominal examination, the Murphy’s sign was positive. She had leukocytosis. Ultrasonography showed features of acute cholecystitis and choledocholithiasis, which was later confirmed by MRI cholangiogram (Fig.1A). ERCP was skipped due to the possible irradiation damage of developing fetus. Emergency combined LC and Lap ECBD was performed. Standard laparoscopic approach for LC was adopted. During the operation, there was gangrenous changes of inflamed gallbladder. (Fig.1B). Cystic duct - common bile duct junction was defined. Dissection and isolation of 1.5 cm cystic duct was performed. Common bile duct exploration through transcystic duct approach was adopted. Choledocholithiasis was removed by endoscopic basket under the guidance of choledochoscopy (Fig.1C, D and E). After ligated and divided the cystic duct and artery, the gallbladder was removed and a drain was placed beside the cystic duct stump. The operation time was 110 min and the blood loss was 30 ml. The drain was removed on postoperative Day 2, the patient recovered well and was discharged on postoperative Day 4. A healthy baby boy was delivered at 40 weeks’ gestation and no developmental problem was noted up to 3 years for the baby delivered.

## 3 Discussion

This report is unique in demonstrating the safety and efficacy of combined LC and lap ECBD for complicated gallstone disease during pregnancy. Complications of gallstones are uncommon in pregnant women, but those could be fatal without prompt treatment. [7] With the advance of modern laparoscopic surgery, many studies have shown that patients can undergo laparoscopic surgery safely in any trimester of pregnancy, without increasing risk to both patients or fetus [[8], [9], [10], [11], [12], [13], [14]]. There was no fetal death reported for LC during the first and second trimester of pregnancy [15]. On the other hand, delaying the necessary surgery may increase the incidence of complications of gallstone disease. As the current standard, LC is regarded as the first choice of treatment for patients with symptomatic gallstone disease during pregnancy [7].

Therapeutic ERCP followed by LC or LC combined with Lap ECBD are the current standard treatment for choledocholithiasis in non-pregnant patients. A meta-analysis involving 1130 patients in eight randomized trials comparing ERCP followed by LC and LC plus Lap ECBD showed similar mortality and complication rates between the two approaches. However, LC plus Lap ECBD was superior in terms of higher stone clearance rate, shorter hospital stay and shorter total operation time [16]. Up till now, there is no randomized study comparing these two approaches in pregnant patients. Although some studies have proven the safety and efficacy of preoperative ERCP in pregnant patients [[17], [18], [19]], the potential risk of ERCP should not be underestimated. In a series of 65 pregnant patients who underwent ERCP [20], 16 percent of patients developed post-ERCP severe pancreatitis and unsatisfactory fetal outcome in patients with first trimester gestation. A national cohort study comparing 58 pregnant women undergone ERCP with a three-fold larger control population of non-pregnant women demonstrated that the rate of post-ERCP pancreatitis was significantly increased in pregnant group (12% *vs.* 5%) (adjusted odds ratio: 2.8) [21].

Fetal ionizing radiation exposure is another concern for ERCP during pregnancy, because of its potential terotogenic effects and subsequent carcinogenesis. To avoid ionizing radiation, a recent trend is performing radiation-free ERCP during pregnancy [22]. In radiation-free ERCP, visualization of bile drainage after wire guided cannulation of common bile duct is used to confirm successful selective biliary cannulation. The disadvantages of this method include the inadvertent cystic duct cannulation by wires or catheters, difficulty in confirmation of common duct cannulation in case of “white bile” in chronically obstructed biliary system, and bile duct injury by curled wires [23].

Favorable clinical outcome of Lap ECBD during pregnancy is rarely reported in the literature. Liberman, et al. [24] reported Lap ECBD in two pregnant patients with choledocholithiasis. In another report by Tuech et al. [25], lap ECBD using trans-cystic duct approach was attempted in a pregnant patient, which was later converted to choledochotomy approach. Kim et al. [26] reported another successful lap ECBD in a pregnant patient who suffered from biliary pancreatitis. All reported patients had normal delivery without major complications or newborn teratogenesis. (Table 1) The current report illustrates another successful lap ECBD using trans-cystic duct approach in pregnancy patient. Regarding the choice of two approach in lap ECBD (trans-cystic or choledochotomy), trans-cystic approach is the preferred initial technique for most patients with small stone (< 10 mm) or normal-sized common bile duct [[27], [28], [29], [30]]. On the contrary, large stone with dilated common duct would be better tackled by choledochotomy approach.

**Table 1:** Case series on laparoscopic exploration of common bile duct during pregnancy.

Study	No. of patients	Trimester			Spontaneous abortion	Preterm	Normal delivery	Approach of common bile duct exploration	
		1^st^	2^nd^	3^rd^				Trans-cystic duct	Choledochotomy
Liberman et al. [24]	2	1	1	0	0	0	2	2	0
Tuech et al. [25]	1	0	1	0	0	0	1	0	1
Kim et al. [26]	1	1	0	0	0	0	1	1	0

## 4 Conclusion

In conclusion, a successful combined LC and LECBD for gangrenous cholecystitis and choledocholithiasis in a pregnant patient is reported. This approach avoids ionizing radiation to the developing fetus, and can cure cholecystitis and choledocholithiasis in one goal during pregnancy.

## Conflict of interest

None of the authors have any commercial or financial involvement in connection with this study that represents or appears to represent any conflicts of interest.

## Sources of funding

This research received no specific grant from any funding agency in the public, commercial, or not-for-profit sectors.

## Ethical approval

This study was exempted from ethnical approval in the Hong Kong University – Shenzhen Hospital.

## Consent

Written informed consent was obtained from the patient for publication of this case report and any accompanying de-identified images. A copy of the written consent is available for review by the Editor-in-Chief of this journal on request.

## Author contribution

Dr Siyuan Qiu performed the surgery and drafted the manuscript. Dr Kelvin Ng participated in the critical revision of the manuscript. Prof. CM Lo, Dr TT Cheung and Dr R Ji were the supervisors. Dr CH Liu, Dr HT Zhu and Dr BR Xu were involved in the management of the patient. All authors read and approved the manuscript.

## Registration of research studies

This is not a research study, registration is not indicated.

## Guarantor

Dr SY Qiu.

Dr Kelvin KC Ng.

## Provenance and peer review

Not commissioned, externally peer-reviewed.
